# Identification of genetic basis of obesity and mechanistic link of genes and lipids in Pakistani population

**DOI:** 10.1042/BSR20180281

**Published:** 2018-07-06

**Authors:** Shabana, Saleem Ullah Shahid, Shahida Hasnain

**Affiliations:** Department of Microbiology and Molecular Genetics, University of the Punjab, Lahore 54590, Pakistan

**Keywords:** Obesity, Polygenic, Pakistan

## Abstract

We aimed to identify the genetic causes of common forms of obesity in the Pakistani people and find out the mechanistic link by observing the relationship of genes and serum lipid traits. Four hundred and seventy-five subjects were genotyped for two mutations in (leptin:N103K and proopiomelanocortin:R236G) and ten common variants in different genes. Serum lipids were also measured. The prevalence of mutations was very low (one heterozygous subject each for both mutations), but fairly high minor/risk allele frequency (M/RAF) was observed for all SNPs. MAF of G2548A was 42.8% in obese and 30.1% in controls (*P*=5.7 × 10^−5^), it showed association with weight, body mass index (BMI), waist circumference (WC), high density lipoprotein cholesterol (HDL-c) and leptin, Gln223Arg had MAF 32% in obese and 18.7% in controls (*P*=5.4 × 10^−6^), it showed association with fasting plasma glucose (FPG) and all lipid traits, Ala54Thr had MAF 42.4% in obese and 33.1% (*P*=0.002), it showed association with none of the tested parameters. rs9939609 MAF was 26.6%, and showed association with none of the tested parameters. rs1802295 (*P*=0.002); rs7178572 (*P*=0.007); rs2028299 (*P*=0.04); rs4812829 (*P*=0.02) showed significant while rs3923113 and rs16861329 did not show a significant association (*P*=0.20 and *P*=0.3, respectively) with obesity. Major genetic contribution to common forms of obesity in Pakistan is from low/modest effect size common variants that act additively to affect body weight quantitatively and mechanism may involve modulating serum lipids.

## Introduction

Obesity, the excess of body fat, has become a global problem. Initially, the explosion occurred in the developed countries due to urbanization, less physical activity, and the use of the high calorie diet but afterward it became a problem of developing countries also [1]. Obesity is a socioeconomic issue as well as a serious predisposing factor to other important medical complications, including diabetes, hypertension, and cardiovascular diseases [2].

Obesity was considered a behavioral disorder previously, but with the observation of familial clustering in obesity, research started on understanding its genetic causes. *Leptin* gene was the first gene in which mutations having severe effects on body weight and satiety regulation were identified. It was followed by identification of mutations in downstream effectors of leptin laying down the basis of monogenic obesity but the recent explosion of obesity could not be explained by these low frequency mutations. The first genome-wide association study (GWAS) in 2007 opened the way to solve the issue and in the same year, three independent GWASs found the same gene, fat mass and obesity associated *(FTO)*, showing association with obesity [3–5]. This discovery laid the ground for the identification of many variants in other non-candidate genes and it is now accepted that common forms of obesity are due to the combined net effect of many risk variants in different genes [6,7].

Pakistan, a low income country, initially faced the problem of malnutrition and under nutrition, but with the improvement in life standards and availability of a variety of palatable foods at relatively cheap price resulted in an increase in obesity which became more evident due to lack of physical activity. Due to illiteracy and psychological stigma, until recently, obesity was not been considered a disease that hindered the research to understand what factors, biochemical as well as genetic, are involved in the development of obesity in Pakistan. With a total population of 184.35 million in 2012–13, Pakistan is the sixth most populous country of the world [8]. According to the Global Burden of Disease Study, in terms of obesity, Pakistan ranked ninth out of 188 countries [9]. Pakistan faced a lot of health challenges during a decade-long war on terrorism [10]. In Asian populations multiple factors increase body fat percentage and resultantly body mass index (BMI) [11] therefore, lower BMI cut-off values have been proposed by the international obesity task force to define overweight (23.0–24.9 kg/m^2^) and obesity (>25.0 kg/m^2^) in Asians [12,13]. According to this criterion, in Pakistan, one-fourth of the general population is either overweight or obese. Various risk factors including female gender, old age, urbanization, and high life standards have been shown to influence body weight significantly [14]. In addition to unhealthy dietary habits, lifestyle changes and decrease in physical activity are the main contributing factors for the increased prevalence of obesity in Pakistan [15]. In this context, it is needed to establish a genetic panel representing the common variants predisposing to the common forms of obesity in this region of the world. There has been limited research in the field of obesity genetics in Pakistan and most of it focussed on monogenic forms. We therefore aimed to look for the possible genetic component responsible for the common forms of obesity. We selected 13 SNPs and 2 mutations in various candidate and non-candidate genes to investigate their association with obesity. We also measured various serum lipid parameters, namely total cholesterol (TC), triglycerides (TG), high density lipoprotein cholesterol (HDL-c), and low density lipoprotein cholesterol (LDL-c) to check the effect of these SNP on these parameters.

## Materials and methods

### Study subjects

The study included a well-characterized and published cohort consisting of a total of 475 subjects (250 cases and 225 controls). Study subject recruitment was done by random sampling from hospitals and general population of Punjab, Pakistan. Subjects signed a written consent and filled in a detailed questionnaire regarding demographic information, lifestyle, exercise habits, and family history of obesity. The inclusion criteria for cases were BMI and waist to hip ratio (WHR) cut offs defined for Asian population previously (BMI > 23 kg/m^2^ as overweight and >26 kg/m^2^ as obese) [2]. Exclusion criteria included pregnancy, presence of malignancies, and recent infections. The study was approved by the institutional ethics committee (Ethical Committee, School of Biological Sciences, University of the Punjab, Pakistan) and all the procedures were carried out in compliance with Helsinki declaration.

### Anthropometric measurements

The measurement of body weight (kg), height (m), waist and hip circumference (HC) (cm) was according to the standard procedures as described previously [17]. BMI (kg/m^2^) and WHR were calculated for each study subject.

### Blood sampling and biochemical analyses

Blood samples were taken after 8–12 h fasting, half sample was used for DNA isolation while the rest half was used to obtain serum. Serum was separated by centrifuging gel vacutainers at 10000 r.p.m. for 10 min, collected in sterilized Eppendorf, and screened for any infectious agents (HBV, HCV, HIV). Any positive samples were discarded and safe samples were used for the lipid profile determination. Serum TC, TG, HDL-c, and LDL-c were measured using commercially available kits (Spectrum Diagnostics, Egypt). Epoch, Biotek microplate reader (BioTek Instruments, Highland Park) was used for all optical density measurements.

### Mutations and SNPs’ selection

The selection was done based on the literature survey identifying the variants which have not been investigated in the Pakistani subjects. We selected both mutations and SNPs in order to find out which cause is more responsible for obesity in Pakistan. The mutations selected were N103K in leptin (*LEP*) gene and R236G mutation in proopiomelanocortin (*POMC*) gene whereas the SNPs were G2548A in leptin (*LEP*) gene, Gln223Arg in leptin receptor (*LEPR*) gene, Ala54Thr in fatty acid binding protein 2 (*FABP2*) gene, rs9939609 in *FTO* gene, rs3923113 near growth factor receptor bound protein (*GRB14*), rs16861329 in sialyltransferase 6 galactosidase 1 protein (*ST6GAL1*), rs1802295 in vacuolar protein sorting associated protein (*VPS26A*), rs7178572 in high mobility group protein 20 A *(HMG20A*), rs 2028299 in adaptor related protein complex (*AP3S2*), and rs4812829 in hepatocyte nuclear factor (*HNF4A*). Although there are >300 loci identified to be associated with obesity, we selected only a small number based on the resources available.

### Genotyping

Genomic DNA was isolated from blood leukocytes using Wizard® Genomic DNA purification kit (Promega, U.S.A.). DNA was quantitated using nanodrop (ND-8000, U.S.A.), and made up to 5 ng/µl concentration. The genotyping methodologies for the mutations and SNPs were based on PCR-RFLP, tetra-ARMS, or TaqMan methods (leptin (*LEP*) gene mutation N103K and SNP G2548A, *LEPR* SNP Gln223Arg, *POMC* mutation R236G, and *FABP2* SNP Ala54Thr, were genotyped by PCR-RFLP method, the *FTO* gene SNP by tetra-ARMS PCR, and rs3923113 near *GRB14*, rs16861329 in *ST6GAL1*, rs1802295 in *VPS26A*, rs7178572 in *HMG20A*, rs 2028299 in *AP3S2*, and rs4812829 in *HNF4A* by TaqMan allelic discrimination assay), the reaction mixture composition and PCR conditions have been described previously [2,17,18,20].

### Statistical analysis

Data analysis was done using the Statistical Package for Social Sciences (IBM SPSS statistics, version 22). Data were analyzed for mean, S.D., and normality of quantitative variables. Quantitative variables showing skewness were log transformed and the results were presented as geometric means with approximate S.D. or S.E.M. The study population was tested for Hardy–Weinberg equilibrium (HWE). Allele/genotype frequencies were calculated and compared between cases and controls using a χ^2^ test. Odds ratio (OR) and 95% confidence intervals were calculated using logistic regression models. Age and gender association with obesity were checked by Mann–Whitney U-test and Pearsons χ^2^ test, respectively, and independent sample *t* tests were used to compare the other continuous variables by obesity. The association of variants with obesity was determined using logistic regression while with anthropometric (BMI, weight, waist and HC, and WHR) and lipid traits was analyzed by analysis of covariance (ANCOVA) with adjustment for age and gender. Linear regression was used to calculate rise/fall in lipid trait levels per risk allele. The analyses were adjusted for potential confounders including age, gender, socioeconomic status, hypertensive, diabetic, CVD status, family history of obesity etc. An online power calculator (https://www.dssresearch.com/KnowledgeCenter/toolkitcalculators/statisticalpowercalculators.aspx) and G Power software were used for power analyses. Due to the inclusion of multiple SNPs, a corrected *P*-value (0.05/10 = 0.005) was used as a significance cutoff.

## Results

The characteristics of the study population have been previously published [2,17,21] and are summarized in Table 1. This population was recruited for the current study only and does not form part of any other study. The proportion of individuals with a family history of obesity, hypertension, cardiovascular problems, and diabetes in obese group is 63.6, 25.5, 12, and 32%, respectively, whereas in controls the values are 8.4, 0.4, 0, and 1.3%, respectively. The diabetic patients were on medication, but the exact information on which medicine is being used could not be traced in the majority of patients due to illiteracy. The number of males and females in the obese group (*n*=250) was 139 (55.6%) and 111 (44.4%), respectively, whereas in the control group (*n*=225) there were 118 (52%) males and 107 (48%) females. There was no association of all variants with age and gender as checked by Mann–Whitney U-test and Pearson’s chi-square, respectively.

**Table 1:** Study population general characteristics

Characteristics	Obese (*n*=250)	Non obese (*n*=225)	*P*-value
Gender:			
Male	139 (55.6%)	118 (52%)	-
Female	111 (44.4%)	107 (48%)	-
Family history	159 (63.6%)	19 (8.4%)	-
Age (years)	39.63 ± 15.19	37.78 ± 11.53	<0.001
Weight (kg)	95.56 ± 16.05	68.63 ± 10.23	<0.001
Height (ft)	5.36 ± 0.43	5.4 ± 0.91	0.536
BMI (kg/m^2^)	34.37 ± 6.08	22.67 ± 5.58	<0.001
WC (cm)	99.55 ± 12.11	71.95 ± 8.65	<0.001
HC (cm)	108.51 ± 12.56	78.23 ± 8.34	<0.001
WHR (WC/HC)	1.00 ± 0.07	0.78 ± 0.01	<0.001
FPG (mg/dl)	102.71 ± 14.12	89.56 ± 7.29	<0.001
TC (mmol/l)	5.63 ± 0.90	4.11 ± 0.79	<0.001
TG (mmol/l)	2.64 ± 0.83	2.12 ± 0.05	<0.001
HDL-c (mmol/l)	1.11 ± 0.10	2.09 ± 0.44	<0.001
LDL-c (mmol/l)	2.57 ± 0.56	2.02 ± 0.34	<0.001
SBP (mmHg)	131.52 ± 5.51	119.52 ± 6.54	<0.001
DBP (mmHg)	83.12 ± 4.91	73.45 ± 5.01	<0.001
Leptin (ng/ml)	21.43 ± 12.24	8.11 ± 2.39	<0.001
Insulin (µU/ml)	18.85 ± 4.13	11.43 ± 5.01	<0.001
HOMA-IR	4.39 ± 3.86	1.95 ± 0.79	<0.001

The table summarizes the general, anthropometric, and biochemical characteristics of the study population. Values are indicated as mean ± S.D. Abbreviations: DBP, diastolic blood pressure; FPG, fasting plasma glucose; HOMA-IR, homeostasis model assessment of insulin resistance; SBP, systolic blood pressure; WC, waist circumference.

### Prevalence of the selected mutations and SNPs

We could detect p. N103K and R236G mutations in heterozygous state each in one subject only in cases and there was no control while having these mutations. The identified subjects were male children with a history of early onset obesity (age of onset: 10 years for p. N103K and <5 years for R236G). The prevalence of mutation in our study was therefore 0.4% in cases, whereas it was not detected in any of the control subjects. The allele and genotype frequencies of the SNPs are shown in Table 2.

**Table 2:** Allele and genotype frequencies of the selected SNPs with respective *P*-values, OR, and confidence interval

SNP	Gene	MAF (%)		Genotype frequency (%)						*P*-value	OR (CI)
		Obese	Non obese	Obese			Non obese				
				Homozygous common	Heterozygous	Homozygous minor	Homozygous common	Heterozygous	Homozygous minor		
G2548A	*LEP*	42.8	30.1	34.8	44.7	20.5	51.1	37.5	11.3	0.028	1.63 (1.07–2.48)
Gln223Arg	*LEPR*	32	18.6	55.2	26	18.8	71.6	19.1	9.3	5.4 × 10^−6^	1.11 (0.98–2.21)
Ala54Thr	*FABP2*	42.4	33.1	35.7	43.4	20.9	50.2	37.6	12.2	0.002	1.56 (0.102–0.364)
rs9939609	*FTO*	35.1	27.5	52.9	35.7	11.5	57.9	37.1	5.0	0.017	2.36 (1.14–4.86)
rs3923113	*GRB14*	24.5	21.9	58.7	33.5	7.8	59.9	36.5	3.7	0.20	0.88 (0.63–1.25)
rs16861329	*ST6GAL1*	23.2	17.8	58.6	36.5	5.0	67.8	28.9	3.3	0.13	0.76 (0.53–1.08)
rs1802295	*VPS26A*	29.4	23	52.5	36.2	11.3	58.1	37.7	4.2	0.02	1.42 (1.03–1.95)
rs7178572	*HMG20A*	52.9	52.1	19.6	53.4	26.9	28.6	38.6	32.9	0.007	1.06 (0.82–1.37)
rs2028299	*AP3S2*	33.2	26.9	47.1	39.5	13.5	52.4	41.4	6.2	0.04	1.33 (0.98–1.81)
rs4812829	*HNF4A*	31	24.8	50.5	37.2	12.4	55.2	40.0	4.8	0.02	1.34 (0.98–1.84)

### Association with the anthropometric and biochemical traits

*LEP* G2548A showed association with weight, BMI, waist circumference (WC), HDL-c, and leptin; Gln223Arg showed association with all lipid traits, Ala54Thr showed association with fasting plasma glucose (FPG) and all lipid traits, rs9939609 showed association with BMI (*P*=0.01), TG (*P*=0.031), LDL-c (*P*=0.03) and a marginal association with TC (*P*=0.059), rs3923113, rs2028299, and rs4812829 showed no association with any of the tested anthropometric and biochemical parameters, rs16861329 showed no association with biochemical traits but a significant association with HC amongst anthropometeric parameters, rs1802295 showed significant association with serum LDL-c, weight, BMI, WC, and HC, rs7178572 showed no association with any of the tested biochemical traits but a significant association with weight, HC, and WHR (Tables 3 and 4).

**Table 3:** *P*-values for association analysis of variants with selected anthropometric parameters tested by logistic regression

SNP	Weight	BMI	WC	HC	WHR
G2548A	4 × 10^−3^	5 × 10^−3^	0.049	0.670	0.448
Gln223Arg	0.002	0.011	0.15	0.15	0.06
Ala54Thr	0.102	0.117	0.120	0.091	0.097
rs9939609	0.084	0.010	0.011	0.919	0.362
rs3923113	0.85	0.41	0.15	0.13	0.92
rs16861329	0.46	0.89	0.09	0.007	0.38
rs1802295	0.0008	0.01	0.01	0.04	0.38
rs7178572	0.02	0.27	0.50	0.01	0.002
rs2028299	0.32	0.26	0.11	0.52	0.10
rs4812829	0.28	0.38	0.07	0.08	0.43

**Table 4:** *P*-values for association analysis of variants with selected biochemical parameters tested by logistic regression

SNP	TC	TG	HDL-c	LDL-c
G2548A	0.077	0.966	0.036	0.383
Gln223Arg	3 × 10^−5^	4 × 10^−2^	3 × 10^−5^	0.036
Ala54Thr	0.031	0.777	0.056	0.035
rs9939609	0.059	0.031	0.083	0.034
rs3923113	0.68	0.77	0.25	0.78
rs16861329	0.47	0.34	0.57	0.36
rs1802295	0.19	0.80	0.06	0.03
rs7178572	0.73	0.89	0.01	0.23
rs2028299	0.22	0.09	0.37	0.60
rs4812829	0.94	0.06	0.09	0.02

## Discussion

Obesity is one of the major public health problems and results from a complex interplay between genes and environmental stimuli. Current efforts to manage obesity have been moderately effective and a better understanding of the development of and the progression to obesity is required for the development of more successful and personalized preventive and therapeutic measures. According to a meta-analysis, the increase in the global burden of obesity can reach upto 57.8% of world’s adult population being overweight or obese by 2030 [22]. Although currently the overall prevalence of overweight and obese individuals is greater in developed countries, the much larger population of the developing countries will result in a considerably larger absolute number of individuals affected by 2030 in the developing world. In addition to larger population size, urbanization, increases in calorie intake, and a reduction in physical activity shall contribute to the epidemic of obesity in the developing regions.

The present study aimed at identifying the probable genetic contribution in the development of obesity in the Pakistani population. The results in the broad sense indicated that in the Caucasians, the research in the field of obesity genetics not only studied the genes involved in the monogenic forms of obesity due to an important role in the energy regulation pathway, but also tried to find out the apparently non-candidate genes, the variants in which can affect the role of candidate genes directly or may modulate certain serum characteristics known to contribute to obesity. On the contrary, the research being carried out in Pakistan emphasized mostly the rare monogenic forms investigating only the candidate genes, there were very few reports of the role of polymorphisms in obesity. We therefore tried not only to compare the prevalence and frequencies of mutations in the candidate genes and variants in the non-candidate genes but also to deduce which one plays a greater role in the progression to obesity in this ethnic group. We thus included 2 mutations and 13 SNPs. We observed marked differences in the prevalence of the mutations (R236G and N103K in the *POMC* and *LEP* genes) and the SNPs. The frequency of mutations was very low as only one individual could be detected for R236G and one for N103K while the minor allele frequencies observed for all SNPs were fairly high (>1%). There has been limited research in the field of obesity genetics in Pakistan and most of it focussed on monogenic forms of obesity but we have shown that common forms of obesity in Pakistan can be explained by the single nucleotide polymorphisms not the mutations, being polygenic in nature whereby common low effect size variants in many genes act quantitatively to affect body weight [2,17,18,20].

The comparison of the minor allele frequencies of the selected polymorphisms showed that the *HMG20A* SNP rs7178572 had the highest minor allele frequency followed by Ala54Thr (*FABP2*), G2548A (*LEP*), rs9939609 (*FTO*), rs2028299 (*AP3S2*), Gln223Arg (*LEPR*), rs4812829 (*HNF4A*), rs1802295 (*VPS26A*), rs9923113 (*GRB14*) and the lowest for rs16861329 (*ST6GAL1*). The differences in allele frequencies are reflected in the location of the SNPs and the functions of the respective genes. *HMG20A* gene encodes a regulatory protein controlling gene expression by histone modification and is important in neural development. The SNP may play its role by affecting the expression of genes involved in lipid metabolism, thereby leading to dyslipidemia and obesity [23,24]. The FTO SNPs are known to affect glucose metabolism and hence lead to obesity [25]. The SNPs resulting in an amino acid change affects the structure and hence the function of protein, which results in aberrant or abnormal protein role.

We found no association of any of the selected variants with age. Only leptin promoter variant G2548A showed a mild association with age, but that could not be relied due to the fact that it is close to the threshold and the analysis should be repeated in order to have reliable results. The observed lack of association may be accounted for possible population stratification, small sample size, and sampling bias. This is in contrast with the previous reports as it is well established that the rate of prevalence of obese or overweight individuals peaks in the age group of 60–69 years [26].

Blood lipid levels are modifiable risk factors for obesity. Lipids constitute a heterogeneous group of biomolecules which are important dietary components and integral part of the cell structure. Being hydrophobic in nature, cholesterol, cholesterol esters, TG, and phospholipids are transported from the liver in the form of lipoproteins. It has been observed that many lipid/lipoprotein abnormalities are prevalent in obesity, such abnormalities are collectively termed as dyslipidemia, however, these dyslipidemias are often hyperlipidemia wherein majority of lipids are shifted toward the upper limits of range or higher than the range. Obesity-associated dyslipidemia is characterized by an increase in TC, TG, LDL-c, and HDL-c, with TG and HDL-c being the most consistent and pronounced. One study considered fat distribution as an important factor for determining differential distribution of TG, HDL-c, and lipoproteins in both sexes and indicated lipid profile in obese persons as an important factor for progression to cardiovascular diseases [27–30]. In the present study, increased TG concentrations were consistently accompanied by low HDL-c concentrations that often coexist with the elevated plasma glucose levels because high amount of sugar in plasma (hyperglycemia) results in the transfer of cholesterol esters from HDL-c to VLDL particles [31]. Further low HDL-c concentration results from its conversion by hepatic lipase into smaller particles which are rapidly cleared from plasma [32]. Resulting VLDL particles form cholesteryl ester depleted small dense LDL-c particles that are taken up by arterial wall macrophages causing atherogenesis, an important sequelae of obesity [33]. However, it is important to note that the fat distribution in the body determines whether good or bad lipids are increased or decreased and resultantly influence the consequences of fat accumulation [34].

The study had some limitations. First, the criterion used to differentiate obese and non-obese subjects was mainly BMI, which is questioned nowadays as a measure of obesity due to the fact that it does not distinguish between fat and lean mass. Other measures which are more accurate for estimating fat mass, e.g. Dual-energy X-ray absorptiometry (DEXA) could not be used due to high cost. Second, in order to draw reliable conclusions, the sample size should be quite large. Due to social and psychological stigmas related to being overweight or obese, a large number of individuals refused to participate in the study. Third, presence of comorbidities in the case group may have confounded certain associations. A large proportion of the obese subjects had a family history of obesity that may have affected the observed results. Although correlation analysis taking into account these comorbidities was performed, it may have affected results to some extent. Another limitation was that the sample population came from the Punjab province only. Although, in the recent past, the social and cultural restrictions have diminished and families marry across provinces in Pakistan, still there are some pooling within castes or tribes. Last, the homogeneity of the population could not be checked by comparing with other populations and the ethnicity was self-decided on the basis of religion, culture, dialect, and homeland, further division into subgroups on the basis of castes was not done.

Future studies with larger sample size and more variants included should be done in order to confirm and validate the reliability of the findings of the current study. Furthermore, if in future, the study sample is derived from all provinces of Pakistan, the results would become more reliable. The implications of the study include providing the baseline information about the role of polymorphisms in the Pakistani population and the probable mechanism through which these variants may exert their effect. However, a more detailed genetic analysis of morbidly obese individuals together with investigation of serum biomarkers elevated or decreased before the onset of clinical forms of obesity should be done in future in order to provide information on the exact mechanism of genotype–phenotype interaction.

## Conclusion

The above work drew some important conclusions about obesity in Pakistan. First, the common, late onset forms involved the low-intermediate effect size variants/single nucleotide polymorphisms in a number of genes that exerted their effect in a quantitative manner, i.e. the common forms of obesity in Pakistan are polygenic in nature. Second, the frequency of mutations selected was very low compared with the modest minor allele frequency of the polymorphisms. Third, the selected polymorphisms did not appear to be associated with age or gender in most of the cases, which may be due to the fact that these may not involve any particular life stage or are not expressed in reproductive tissues or affect sexual hormones or characteristics. Fourth, the selected variants significantly affected many anthropometric traits, notably weight and BMI which indicated a role of these variants in fat deposition. Last, we observed a consistent relationship of most of the variants on lipid profile which showed that the progression to obesity in subjects carrying the risk alleles may involve disturbing the lipid parameters, although its not clear until now that whether it is the aberrant lipid profile that leads to obesity or it is the presence of excess trans fats in obese subjects that results in derangement of the lipid profile. In future, a genetic panel consisting of common variants in addition to a routine biochemical assessment of serum should provide better risk prediction for predisposition to obesity in high risk individuals.

## Abbreviations

AP3S2: adaptor related protein complex
BMI: body mass index
CDV: cardiovascular disease
FABP2: fatty acid binding protein 2
FTO: fat mass and obesity associated
GRB14: growth factor receptor bound protein
GWAS: genome-wide association study
HBV/HCV: hepatitis B/C viruses
HC: hip circumference
HDL-c: high density lipoprotein cholesterol
HMG20A: high mobility group protein 20 A
HNF4A: hepatocyte nuclear factor
LDL-c: low density lipoprotein cholesterol
MAF: minor allele frequency
PCR-RFLP: polymerase chain reaction-restriction fragment length polymorphism
SNP: single nucleotide polymorphism
ST6GAL1: sialyltransferase 6 galactosidase 1 protein
TC: total cholesterol
TG: triglyceride
VPS26A: vacuolar protein sorting associated protein
VLDL: very low density lipoprotein
WC: waist circumference
WHR: waist to hip ratio
